# The development of a decision aid to support treatment choice in pelvic organ prolapse: a Delphi study

**DOI:** 10.1186/s12911-025-03209-y

**Published:** 2025-10-14

**Authors:** Larissa Esmeralda Drost, Marjan Stegeman, Janneke van Dijk, Romy E. D. Lamers, Regina The, Maria B. E. Gerritse, Arie Franx, M. Caroline Vos

**Affiliations:** 1 Department of Obstetrics and Gynaecology, Erasmus Medical Centre, PO Box 2040, Rotterdam, 3000 CA the Netherlands; 2 Department of Obstetrics and Gynaecology, Elisabeth-Tweesteden Hospital, PO Box 90151, Tilburg, 5000 LC the Netherlands; 3 Department of Urology, UMC Utrecht, PO Box 85500, Utrecht, 3508 GA the Netherlands; 4 ZorgKeuzeLab, Gasthuisplaats 1, Delft, 2611 BN The Netherlands; 5 Department of Obstetrics and Gynaecology, Gelderse Vallei Hospital, PO box 9025, Ede, 6710 HN the Netherlands

**Keywords:** Decision aid, Decision support techniques, Delphi procedure, Patient participation, Pelvic organ prolapse, Shared decision making.

## Abstract

**Background:**

Pelvic organ prolapse is a lifelong condition which affects the quality of life. It can be treated in different ways, but there is no evidence-based optimal treatment. Treatment options are pelvic floor muscle training, a pessary or surgery and treatment choice should depend on patient preference. The aim of this study is to develop a web-based decision aid to support treatment choice in patients with symptomatic pelvic organ prolapse.

**Methods:**

For the development of this decision aid an online Delphi consensus procedure was performed. Patients and gynaecologists rated their (dis)agreement concerning a list of statements using 5-point-likert scales. Value clarification exercises were included in the decision aid to help patients become more aware of their values. After the development of the decision aid using the results of the Delphi consensus procedure, a usability test for both patients and clinicians was performed.

**Results:**

Patients reached consensus in 11 out of 13 statements (85%). Clinicians reached consensus in 38 out of 44 statements (86%). The usability test yielded positive feedback regarding the comprehensibility, usability, images, the amount of information provided and the developed value clarification exercises. Both patients and clinicians showed strong preference for the use of a decision aid in the decision-making process.

**Conclusions:**

This decision aid was developed to support patients with symptomatic pelvic organ prolapse in the process of decision-making. A usability test with both patients and gynaecologist yielded positive feedback. The development of this online decision aid is a first step to improving the care for women with symptomatic pelvic organ prolapse.

**Trial registration:**

NL 55737.028.15, 30/10/16.

**Supplementary Information:**

The online version contains supplementary material available at 10.1186/s12911-025-03209-y.

## Background

Pelvic organ prolapse (POP) is a common problem among women worldwide. As a consequence of POP, 8.7% of Dutch women between the age of 45–84 experience complaints [1]. Due to POP, women experience a high prevalence of urinary, prolapse, and defecatory symptoms [1, 2]. Even though POP is a benign condition, without treatment it can cause lifelong symptomatic distress and result in an impaired quality of life [3]. Treatment options known effective for POP are pelvic floor muscle training (PFMT) (for minor stages of POP), pessary treatment and prolapse surgery [4–7]. Recently a randomized controlled trial has been performed to compare the efficacy of a pessary to surgery. Even though none of the treatment options was superior, the patient reported subjective improvement was high in both groups, as is also seen in other comparative studies [8–10]. With no treatment option being superior, current Dutch guidelines recommend to discuss all treatment options with the patient [11]. 

Decision-making with regard to treatment choices is affected by many factors. Therefore, careful exploration of preferences and expectations is needed before deciding on a treatment option [12]. In recent years, more attention is given to the involvement of the patient perspective in decision-making, a process which is known as shared decision making (SDM) [13]. Especially in situations when there is no proven clinically preferred treatment option, SDM results in better affective and cognitive outcomes [13]. 

A decision aid (DA) aims to support SDM and involve patients in the decision procedure. In general, it makes patients feel more knowledgeable, better informed and clearer about their values [14]. They have a more active role in decision-making, more accurate risk perceptions and experience lower decisional conflict without an increase in anxiety levels [14, 15] To design such a DA, a Delphi consensus procedure can be used. This technique is designed to transform individual opinions into group consensus and is mainly used in situations where a new concept needs to be built from scratch [16]. 

On the development of an online DA for women with symptomatic POP who have to choose between different treatment options (and who have not yet decided to undergo prolapse surgery) has only been reported in one other study [17]. Therefore, the primary objective of this study was to develop a DA to support treatment choice in patients with symptomatic POP and achieve consensus among patients and clinicians about the content of this DA. Next to this, we wanted to explore whether the involved patients believe this DA would be helpful and whether gynaecologists are motivated to use the DA in future consultations.

## Methods

### Study design

We conducted an online Delphi consensus procedure to achieve consensus among selected experts (patients and clinicians) of the Delphi panel about the content of a DA for the treatment of POP. We developed the DA in accordance with the International Patient Decision Aid Standards (IPDAS) [18, 19]. For the online questionnaire rounds the ‘Qualtrics: Online Survey Software & Insight Platform’, was used. The study was approved by the Medical Ethical Research Committee Brabant, Tilburg, the Netherlands (NW 2015-62). The trial was registered as the SHAred DEcision making in Pelvic Organ Prolapse (SHADE-POP) trial, NL 55737.028.15.

### Participants

Patients were recruited through the gynaecologic patient association (*Bekkenbodem4all*) in the Netherlands. A message containing information on the survey was posted on Facebook and shared on the homepage of *Bekkenbodem4all*. These messages were also shared by the research team among their patients. The included patients self-reported to be currently diagnosed with POP by either a general practitioner or a gynaecologist or suffered from POP in the past. Both patients with and without pessary and/or surgery in the past were included. Exclusion criteria were not having access to the internet and not having sufficient knowledge of the Dutch language. Patients were offered a patient information letter and had to sign an informed consent form.

We recruited gynaecologists employed in Dutch hospitals and specialized in urogynaecology or the pelvic floor through the Dutch Urogynaecological Association. We contacted them by e-mail and asked for their participation in the online questionnaire. The e-mail provided information on the aim and design of the study, the Delphi procedure and an estimation of the time needed for participation. A subsequent e-mail followed, containing a personalized web-link to the Qualtrics website where the Delphi questionnaire could be accessed.

As there are no clear guidelines for the required number of participants in a Delphi consensus procedure (generally 10–15 subjects are reported), we aimed for a minimum of 10 patients and 10 gynaecologists. The Delphi procedure was performed in 2015.

### Questionnaire round

During the questionnaire round, the expert panel was presented a set of statements and open comment fields. The statements were developed by the research team through a combination of the expert knowledge on the subject of several gynaecologists with extensive experience in the care for women with POP, and the requirements for a DA as stated in the IPDAS criteria [18]. These requirements state, among other criteria, which aspects of the health condition, treatment options and their outcomes should be provided and how they should be presented. Participants were asked to indicate the importance of each of the provided statements using a 5-point Likert scale, ranging from 1 (not important) to 5 (very important).

For the patients, demographic characteristics as shown in Table 1 were obtained. These were followed by 13 statements in two different categories: (1) Information provision and (2) Decision aid. Patient questionnaires also contained several open-ended questions. Open comment fields were used to allow participants to mention additional statements or items which they consider important but were not included in the listed statements.

For the clinicians, demographic characteristics were also obtained, as shown in Table 2. After this, 44 statements were presented in four different categories: (1) Decision aid, (2) Usability and applicability, (3) Risks and side effects and (4) Value clarification exercises (VCEs). Open comment fields were provided for additional statements or items not included in the statements. Moreover, the questionnaire also contained eleven open questions regarding expected advantages and disadvantages of the DA and several treatment options to be included with their specific advantages and disadvantages.

The content for the draft of the DA was selected by the research team based on results from the Delphi procedure and guidelines for the development of a DA [18]. From these results, the research team also drafted the final formulation of the VCEs. The final text for the DA including the VCEs was shared between the research team members until consensus was reached.

### Usability test

After the design of the DA, a usability test was performed to get an impression of the DA before testing it on a larger scale. Four patients were asked for participation by their gynaecologist during an outpatient visit. They were requested to go through the entire DA in the presence of two researchers (JD and RT), who are not involved in the treatment of the patients. Before the patients started, they were asked to think out loud and point out all impressions during the process. After finishing the DA, additional questions were asked concerning first impression, positive and negative aspects, feelings experienced while going through the DA and whether or not they would recommend the DA to other patients.

A usability test was also performed with three gynaecologists in the presence of the two researchers. They were asked to go through the DA the same way the patients did, with particular attention for the medical content of the DA. After finishing, additional questions concerning first impression, positive and negative aspects and their attitude towards the DA were asked. During the usability test of both the patients and the gynaecologists notes were taken by the two researchers.

Both patients and gynaecologists who participated in the usability test were not involved in the Delphi panel nor part of the research team. The current form of the DA in Dutch can be found online [20].

### Data analysis

Based on a 5-point Likert scale, the outcomes were classified by using median scores. Consensus on agreement of a statement was defined as a median score of 4 or higher [16, 21]. Consensus on disagreement of a statement was defined as a median score lower than 3. No consensus was defined as a median of 3.

The results of the usability test were used to make (minor) adjustments to the final version of the DA.

## Results

### Participants

Sixteen patients were recruited with a median age of 57.5 years (Table 1). Several patients received more than one treatment option subsequently. Eight (50%) patients indicated they received most information from their gynaecologist. Only one (6%) patient mentioned the patient leaflet and three (19%) patients mentioned the internet. The response rate of the invited gynaecologists for the questionnaire round was 46% (*N* = 11). The median age for the gynaecologists was 46 years, with a median of 18.5 years of clinical experience (Table 2).

**Table 1:** Patient characteristics

Patient characteristics	Median or *N* (%)
Age	57.5
**Educational level** ^**a**^	
Low	0
Medium	7 (44)
High	9 (56)
**Time since treatment decision**	
< 1 year	6 (38)
1–2 years	3 (19)
2–5 years	2 (13)
5–10 years	4 (25)
> 10 years	1 (6)
**Received treatments**	
None	2 (13)
Pelvic floor muscle training	9 (56)
Pessary	8 (50)
Surgery	6 (38)
**Main source of information**	
Gynaecologist	8 (50)
Internet	3 (19)
Patient information leaflet	1 (6)
Other^b^	4 (25)
**Satisfaction with treatment choice**	
Yes	15 (94)
No	1 (6)

^a^ Low (none or primary school only), medium (lower general secondary education or vocational education), high (pre-university education, high vocational education, university)

^b^ General practitioner, pelvic floor muscle therapist, patient association or combination of different clinicians

**Table 2:** Gynaecologist characteristics

Gynaecologist characteristics	Median or *N* (%)
Age	46
Years of clinical experience	18.5
Prolapse patients per year per expert	500
Prolapse surgery at clinic per year	183.5
**Gender**	
Female	7 (64)
Male	4 (36)

### Questionnaire round patients

For the patients, the questionnaire contained 13 statements, divided in two categories: (1) Information provision and (2) Decision aid. The agreement to each of the statements, as provided to the patients, is presented in supplementary file 1. The patients reached consensus on 11/13 statements (85%).

Consensus among patients concerned the following aspects. Patients were satisfied with the content and amount of the provided information and felt like they had participated in the decision-making process around their own treatment for POP. Furthermore, patients agreed that they would review the information in the DA even if it would take effort and time. They agreed that they should be allowed to skip specific parts of the provided information, i.e. a treatment option which is not suitable in their case. No consensus was reached regarding the knowledge of the risks and side effects of a pessary and expectant management. This should be interpreted as a considerable difference in knowledge of the patients on these treatment options and therefore these subjects were included in the DA. The open ended questions and comment fields were mainly used by the patients to confirm the already mentioned options and elaborate on their personal situation. Accuracy of the provided information and clarity were mentioned as important.

### Questionnaire round gynaecologists

The questionnaire for the gynaecologists contained 44 statements, divided in four categories: (1) Decision aid, (2) Usability and applicability, (3) Risks and side effects and (4) Value clarification exercises. The agreement on each of the statements, as provided to the gynaecologists, is presented in supplementary file 2. Consensus was reached on 38 of the 44 statements (86%).

Gynaecologists found it important to offer the DA to any patient who has to make a treatment decision and agreed on the treatment options which should be included. There was consensus that the DA should help to clarify patients’ preferences, end in a summary and should allow patients to skip specific parts of the provided information. No consensus was reached on the two statements regarding whether or not the DA should give a treatment advice. Therefore, a treatment advice was not included in the DA.

In the category ‘risks and side effects’ agreement was reached on 7/9 statements concerning a pessary. No consensus was reached on mentioning the daily confrontation with the pessary and whether the risks and side effects that occur in less than 1% of patients should be mentioned. Hence, these items were not included in the DA. Concerning POP surgery, the gynaecologists agreed on all six statements.

In the category ‘VCEs’ consensus was reached on all statements, with 7/8 being a positive consensus. Negative consensus was obtained on a VCE on the use of a pessary in a situation in which it might not be necessary. As well as the patients, the clinicians agreed that it should be allowed to skip parts of the provided information.

The comments given in the open comment fields mainly focused on whether expectant management should be mentioned as an option in the DA. Some gynaecologists state that it cannot be seen as a treatment option and should therefore not be included in the DA. However, other gynaecologists explicitly advised to mention the option as some patients with POP visit a physician as a precaution, and not because they experience any problems. It was also suggested to explain that PFMT can be combined with other treatment options. Furthermore, additional evidence from literature concerning a few statements was added in the open comment fields.

Originally, we planned to perform a second round of the Delphi consensus procedure. However, due to high level of consensus in both the patient expert and the clinical expert group in the first round, we concluded that saturation was reached, and a second round would not reveal extra information.

**Fig. 1:**
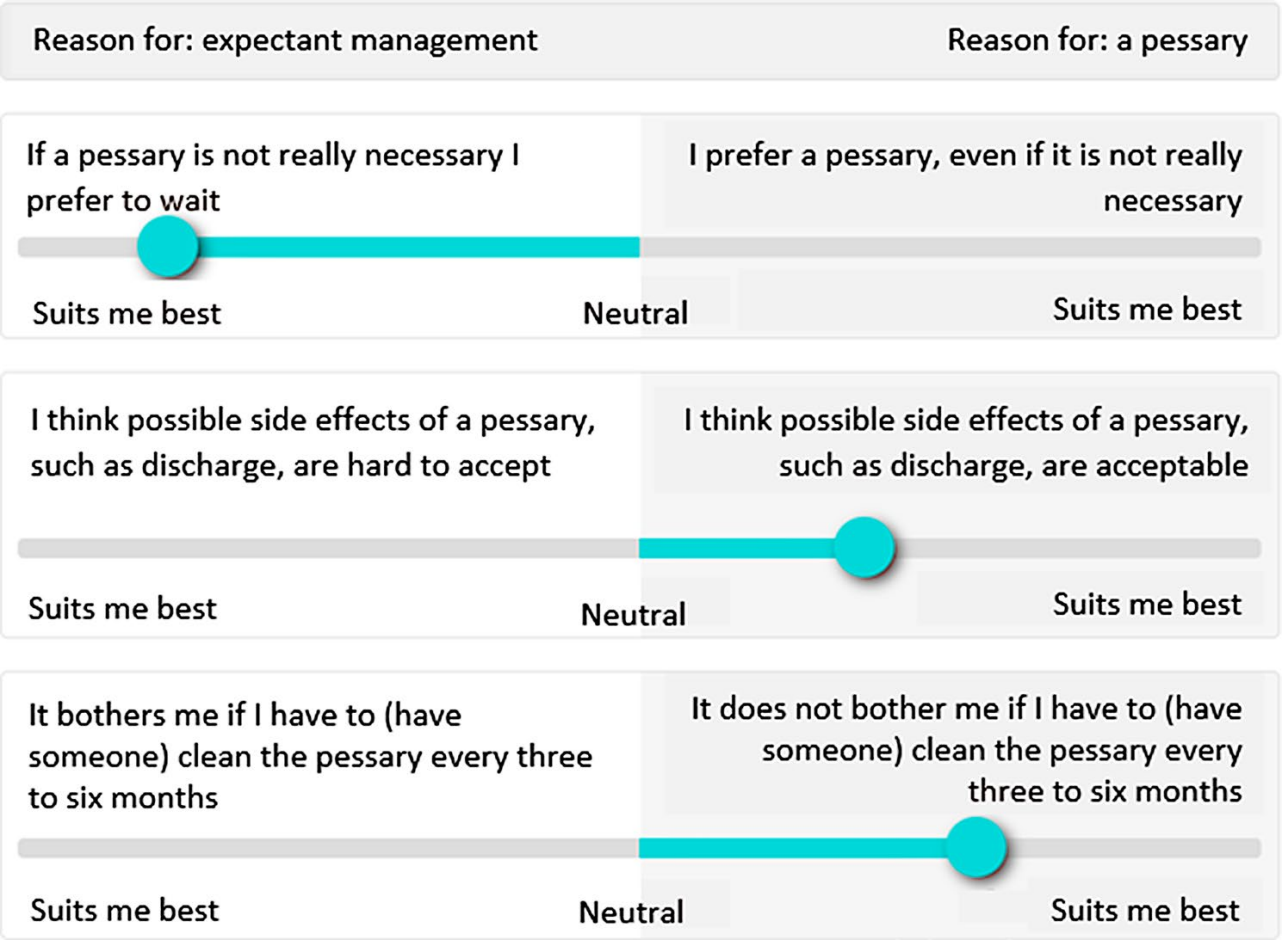
Example of VCE

Next, the DA was developed containing different sections on the selected topics. The first section focuses on the causes and different types of POP, whether treatment is necessary and the possible treatment options in general. In the second section, the patient is requested to indicate which treatment options have been mentioned as possible options to her. This information is then used to create a third section containing information on expectant management and a pessary, or a fourth section containing information on a pessary and surgery. For all sections, if available, yet developed informative texts from the scientific and patient association were used. This information was already checked on comprehensibility for patients with different characteristics. Both sections three and four contain VCEs, of which an example can be seen in Fig. 1 or online [20]. The fifth section provides a summary of the preferences of the patient by presenting the results of the VCEs.

### Usability test

Four patients with experience in making a treatment choice regarding POP were asked for participation during outpatient visits. Recurring themes were extracted by the researchers present during the usability test. All patients indicated a DA would have been helpful during their decision-making and would recommend the developed DA to other patients. Positive aspects of the DA were the images, understandable language, use of VCEs and the summary at the end. Information regarding prolapse and sexuality was missed and sometimes language was experienced as too difficult to understand. The language marked as too complex by the patients was adjusted in the final version of the DA.

In addition, three gynaecologists tested the DA. They also mentioned design, images, VCEs and the summary as positive aspects and would all like to use the DA in the future. Like the patients, they noted a lack of information concerning sexuality. Furthermore, they pointed out that for extra emphasis a paragraph about lifestyle should be placed both at the beginning and the end of the DA. As both patients and gynaecologists mentioned the lack of information concerning sexuality and prolapse, a short paragraph on this matter was added to the final version of the DA.

## Discussion

Using a Delphi procedure, we developed a DA for patients with symptomatic POP. A high rate of consensus was found for both patients and clinicians about the DA and information provision. Both groups were positive about the comprehensibility of the DA, usability, images, amount of information and the VCEs.

After finishing the DA, patients must have been helped to understand treatment options, their potential benefits and possible side effects [22]. DAs have been developed to facilitate this process and have shown to increase patient understanding and assist patients in the process of SDM [23]. Many of these studies used a Delphi consensus procedure for development as well, with positive results [18, 24]. The Delphi consensus procedure for our DA resulted in a DA providing information on POP and the treatment options including benefits and possible side effects. As a result of the high consensus rate in both the clinical expert and the patient expert group, we decided to perform only one round of the Delphi procedure. Because of the high consensus rate, we expect our DA to be able to assist patients and increase patient understanding as well.

So far, many DAs for other conditions like breast cancer, benign prostatic hyperplasia and several psychologic disorders have been developed. Two DAs on POP have been reported on but they have remained nonvalidated [17, 25]. Even though POP is a benign condition, it will last lifelong when left untreated and can cause symptomatic distress. As there is no optimal treatment option and all treatment options can have complications, and an effect on quality of life, it underscores the importance of paying attention to patient preferences.

POP surgery has a recurrence rate of up to 30% in the first two years [7]. Keeping this high rate in mind, many clinicians prefer their patients to try PFMT and/or a pessary first. In general, DAs are known to increase the accuracy of risk perceptions and favour the choice of more conservative options, resulting in less patients having surgery as preferred treatment option [14]. Therefore, a DA for POP may result in patients preferring a less invasive treatment option.

### Strengths

A strength of this study is the use of the Delphi method. The Delphi method is a well-known method for exploring views of experts on subjects of which little is known in advance [16]. The use of patients with experience around decision-making for POP as well as experienced clinicians ensured that the content, process of development and the efficiency of the online tool are representative for new patients. The availability of open comment fields assured that no important content would be missed. However, the aspect of sexuality only came forward in the usability test.

A valuable component of this DA for POP are the VCEs which provide insight in the values individuals place on the consequences of the different treatment options. They typically list the benefits, harms and uncertainties of the options and encourage patients to consider which of these are most important to them [26]. The use of VCEs as part of a DA is known to result in a higher proportion of patients choosing an option congruent with their values and is likely to be associated with positive patient outcomes [14, 27]. Literature also shows that a high amount of treatment options may have a negative influence on the rate of VCE completion [26]. As there are only three treatment options for POP, VCEs are expected to be a helpful addition to this DA.

### Limitations

A limitation of the study might be the participation rate of the invited gynaecologists. After explaining the Delphi procedure and two reminder e-mails, only 46% of the invited gynaecologists participated. As our questionnaire was extensive and took time and effort to complete, the main reason for the drop-out was lack of time. To ensure the quality of the DA, we did not shorten the questionnaire. Our participation rate of 46% is comparable with other studies for the development of a DA [24, 28]. 

We originally planned to perform two Delphi rounds. Regarding the number of rounds performed, literature shows great variation. In our study the items for the questionnaire round were generated by several experienced gynaecologists, with extensive experience in guiding treatment decisions around POP, whereas in other Delphi studies the first round of questionnaires is often used for this purpose [29]. Items were selected as described in the IPDAS criteria and included providing information about options, presenting probabilities and clarifying and expressing values [18]. Due to the high degree of consensus in both expert groups, we decided that we had reached data saturation in one Delphi round. As a second Delphi round would require extensive effort from the expert groups again, we decided to cancel the second round.

Another limitation of the DA might be that it is only available online. However, electronically delivered SDM interventions might be associated with even more positive patient outcomes [27]. The age of the patients participating in the Delphi study was comparable to the average age of the POP population with an average of 57 years [1]. The educational level of the selected patients was relatively high as the selection through the patient association might have caused bias. However, it is unknown whether higher age and educational level will have an effect on the use of the DA in practice. These patient characteristics might result in limited generalizability of the DA. The advantage of a DA with online availability is the possibility for patients to use it anywhere regardless of the setting of care or the presence of a care professional.

## Conclusion

A Dutch online DA was developed to support patients with symptomatic POP in the process of decision-making. A Delphi method was used to include the expertise of patients and clinicians and to optimize the DA to the needs of the target population. A usability test with both patients and gynaecologists yielded positive feedback and resulted in minor adjustments. By including both patients and clinicians, we aimed to increase the quality of the DA and facilitate quick implementation. The development of this online DA is a first step to improving the care for women with symptomatic POP. A cluster randomized trial is currently performed to evaluate the effect of the DA on SDM and information provision [30]. 

## Supplementary Information

Below is the link to the electronic supplementary material.

Supplementary Material 1

Supplementary Material 2

## Data Availability

The datasets used and/or analysed during the current study are available from the corresponding author on reasonable request.

## Abbreviations

DA: Decision aid
IPDAS: International Patient Decision Aid Standards
PFMT: Pelvic floor muscle training
POP: Pelvic organ prolapse
SDM: Shared decision making
VCE: Value clarification exercise

## References

[1] Slieker-ten Hove MC, Pool-Goudzwaard AL, Eijkemans MJ, Steegers-Theunissen RP, Burger CW, Vierhout ME. The prevalence of pelvic organ prolapse symptoms and signs and their relation with bladder and bowel disorders in a general female population. Int Urogynecol J Pelvic Floor Dysfunct. 2009;20(9):1037–45. 19444368 10.1007/s00192-009-0902-1 PMC2721135

[2] Kapoor DS, Thakar R, Sultan AH, Oliver R. Conservative versus surgical management of prolapse: what dictates patient choice? Int Urogynecol J Pelvic Floor Dysfunct. 2009;20(10):1157–61. 19543676 10.1007/s00192-009-0930-x

[3] Chan SS, Cheung RY, Yiu KW, Lee LL, Pang AW, Chung TK. Symptoms, quality of life, and factors affecting women’s treatment decisions regarding pelvic organ prolapse. Int Urogynecol J. 2012;23(8):1027–33. 22398825 10.1007/s00192-012-1698-y

[4] Barber MD, Walters MD, Bump RC. Short forms of two condition-specific quality-of-life questionnaires for women with pelvic floor disorders (PFDI-20 and PFIQ-7). Am J Obstet Gynecol. 2005;193(1):103–13. 16021067 10.1016/j.ajog.2004.12.025

[5] Lamers BH, Broekman BM, Milani AL. Pessary treatment for pelvic organ prolapse and health-related quality of life: a review. Int Urogynecol J. 2011;22(6):637–44. 21472447 10.1007/s00192-011-1390-7 PMC3097351

[6] Li C, Gong Y, Wang B. The efficacy of pelvic floor muscle training for pelvic organ prolapse: a systematic review and meta-analysis. Int Urogynecol J. 2016;27(7):981–92. 26407564 10.1007/s00192-015-2846-y

[7] Maher C, Feiner B, Baessler K, Christmann-Schmid C, Haya N, Brown J. Surgery for women with anterior compartment prolapse. Cochrane Database Syst Rev. 2016;11:CD004014. 27901278 10.1002/14651858.CD004014.pub6 PMC6464975

[8] Cheung RY, Lee JH, Lee LL, Chung TK, Chan SS. Vaginal Pessary in women with symptomatic pelvic organ prolapse: a randomized controlled trial. Obstet Gynecol. 2016;128(1):73–80. 27275798 10.1097/AOG.0000000000001489

[9] Coolen AWM, Troost S, Mol BWJ, Roovers J, Bongers MY. Primary treatment of pelvic organ prolapse: pessary use versus prolapse surgery. Int Urogynecol J. 2018;29(1):99–107. 28600758 10.1007/s00192-017-3372-x PMC5754400

[10] van der Vaart LR, Vollebregt A, Milani AL, Lagro-Janssen AL, Duijnhoven RG, Roovers JWR, et al. Effect of Pessary vs surgery on Patient-Reported improvement in patients with symptomatic pelvic organ prolapse: a randomized clinical trial. JAMA. 2022;328(23):2312–23. 36538310 10.1001/jama.2022.22385 PMC9857016

[11] Prolaps. 2014 [Available from: https://richtlijnendatabase.nl/richtlijn/prolaps/prolaps_-_startpagina.html.

[12] Basu M, Wise B, Duckett J. A qualitative study of women’s preferences for treatment of pelvic floor disorders. BJOG. 2011;118(3):338–44. 21134102 10.1111/j.1471-0528.2010.02786.x

[13] Shay LA, Lafata JE. Where is the evidence? A systematic review of shared decision making and patient outcomes. Med Decis Mak. 2015;35(1):114–31. 10.1177/0272989X14551638 PMC4270851 25351843

[14] Stacey D, Legare F, Lewis K, Barry MJ, Bennett CL, Eden KB, et al. Decision aids for people facing health treatment or screening decisions. Cochrane Database Syst Rev. 2017;4:CD001431. 28402085 10.1002/14651858.CD001431.pub5 PMC6478132

[15] Knops AM, Legemate DA, Goossens A, Bossuyt PM, Ubbink DT. Decision aids for patients facing a surgical treatment decision: a systematic review and meta-analysis. Ann Surg. 2013;257(5):860–6. 23470574 10.1097/SLA.0b013e3182864fd6

[16] Hasson F, Keeney S, McKenna H. Research guidelines for the Delphi survey technique. J Adv Nurs. 2000;32(4):1008–15. 11095242

[17] Hulbaek M, Knutz E, Ebbesen NT, Primdahl J, Nielsen JB, Birkelund R. Pelvic organ prolapse and treatment decisions- developing an online preference-sensitive tool to support shared decisions. BMC Med Inf Decis Mak. 2020;20(1):265. 10.1186/s12911-020-01264-1 PMC7565805 33059668

[18] Elwyn G, O’Connor A, Stacey D, Volk R, Edwards A, Coulter A, et al. Developing a quality criteria framework for patient decision aids: online international Delphi consensus process. BMJ. 2006;333(7565):417. 16908462 10.1136/bmj.38926.629329.AE PMC1553508

[19] Joseph-Williams N, Newcombe R, Politi M, Durand MA, Sivell S, Stacey D, et al. Toward minimum standards for certifying patient decision aids: a modified Delphi consensus process. Med Decis Mak. 2014;34(6):699–710. 10.1177/0272989X13501721 23963501

[20] Verzakking keuzehulp: R. The. 2018 [Available from: https://verzakking.keuzehulp.nl/over-keuzehulp

[21] Lamers LM, McDonnell J, Stalmeier PF, Krabbe PF, Busschbach JJ. The Dutch tariff: results and arguments for an effective design for National EQ-5D valuation studies. Health Econ. 2006;15(10):1121–32. 16786549 10.1002/hec.1124

[22] Thistlethwaite J, Evans R, Tie RN, Heal C. Shared decision making and decision aids - a literature review. Aust Fam Physician. 2006;35(7):537–40. 16820831

[23] Feldman-Stewart D, Brennenstuhl S, McIssac K, Austoker J, Charvet A, Hewitson P, et al. A systematic review of information in decision aids. Health Expect. 2007;10(1):46–61. 17324194 10.1111/j.1369-7625.2006.00420.x PMC5060377

[24] Lamers RED, Cuypers M, Garvelink MM, de Vries M, Bosch J, Kil PJM. Development of a decision aid for the treatment of benign prostatic hyperplasia: a four stage method using a Delphi consensus study. Patient Educ Couns. 2016;99(7):1249–56. 26899631 10.1016/j.pec.2016.02.004

[25] Brazell HD, O’Sullivan DM, Forrest A, Greene JF. Effect of a decision aid on decision making for the treatment of pelvic organ prolapse. Female Pelvic Med Reconstr Surg. 2015;21(4):231–5. 25521472 10.1097/SPV.0000000000000149

[26] Peate M, Watts K, Wakefield CE. The ‘value’ of values clarification in cancer-related decision aids. Patient Educ Couns. 2013;90(2):281–3. 23194822 10.1016/j.pec.2012.10.023

[27] Mathijssen EGE, van den Bemt BJF, van den Hoogen FHJ, Popa CD, Vriezekolk JE. Interventions to support shared decision making for medication therapy in long term conditions: a systematic review. Patient Educ Couns. 2020;103(2):254–65. 31493959 10.1016/j.pec.2019.08.034

[28] Mokkink LB, Terwee CB, Patrick DL, Alonso J, Stratford PW, Knol DL, et al. The COSMIN study reached international consensus on taxonomy, terminology, and definitions of measurement properties for health-related patient-reported outcomes. J Clin Epidemiol. 2010;63(7):737–45. 20494804 10.1016/j.jclinepi.2010.02.006

[29] Gracht HAvd. Consensus measurement in Delphi studies: review and implications for future quality assurance. Technol Forecast Soc Change. 2012;79(8):1525–36.

[30] Drost LE, Stegeman M, Gerritse MBE, Franx A, Vos MC, group S-Ps. A web-based decision aid for shared decision making in pelvic organ prolapse: the SHADE-POP trial. Int Urogynecol J. 2023;34(1):79–86. 10.1007/s00192-022-05405-0 PMC9665015 36378318

