# Supplementary material for: The development of a decision aid to support treatment choice in pelvic organ prolapse: a Delphi study
Source: BMC Med Inform Decis Mak. 2025 Oct 14;25:380. doi: 10.1186/s12911-025-03209-y (PMC12522796; doi:10.1186/s12911-025-03209-y)
Supplement: Supplementary file 1 — Supplementary Material 1 [file 12911_2025_3209_MOESM1_ESM.docx]

**Questionnaire round among patients**

| **Questions** | **Median** | **Consensus** |
| --- | --- | --- |
| **Information provision (N=8)** |  |  |
| 1. I am satisfied with the information, about my prolapse, the gynaecologist has provided me | 4 | Yes |
| 1. I preferred more information about my treatment options | 2 | Yes |
| 1. I felt that I had no participation in the choice of treatment | 1 | Yes |
| 1. I did not know that there were several treatment options possible | 1 | Yes |
| 1. I was aware of the side effects / complications of expectant management | 3 | No |
| 1. I was aware of the side effects / complications of a pessary | 3 | No |
| 1. I was aware of the side effects / complications of a surgery | 4 | Yes |
| 1. I would have liked to have more time for making the treatment decision | 2 | Yes |
| **Decision aid (N=5)** |  |  |
| 1. I want to review the information in this decision aid, even if it costs me time and effort | 5 | Yes |
| 1. It is no problem spending more than half an hour going through a decision aid | 5 | Yes |
| 1. If the decision aid involves several steps, patients may choose to skip steps/information | 4 | Yes |
| 1. The decision aid should give a treatment advice based on patients answers | 4 | Yes |
| 1. The decision aid should help patients to define a treatment preference but should not give a treatment advice | 4 | Yes |
