# Supplementary material for: The development of a decision aid to support treatment choice in pelvic organ prolapse: a Delphi study
Source: BMC Med Inform Decis Mak. 2025 Oct 14;25:380. doi: 10.1186/s12911-025-03209-y (PMC12522796; doi:10.1186/s12911-025-03209-y)
Supplement: Supplementary file 2 — Supplementary Material 2 [file 12911_2025_3209_MOESM2_ESM.docx]

**Questionnaire round among gynaecologists**

| **Questions** | **Median** | **Consensus** |
| --- | --- | --- |
| **Decision aid (N=5)** |  |  |
| The decision aid should be offered to: |  |  |
| 1. All patients with a symptomatic prolapse | 4 | Yes |
| 1. All patients with a prolapse in which a wait and see treatment (which included pelvic floor physiotherapy) is not effective | 4 | Yes |
| 1. All patients with a prolapse in which a pessary is not effective | 4 | Yes |
| 1. Only patients in which treatment with a pessary and wait and see treatment is not effective | 4 | Yes |
| 1. Patients who already had surgery for their prolapse and have a recurrent prolapse | 4 | Yes |
| **Usability and applicability (N=14)** |  |  |
| 1. The decision aid should give the option ‘wait and see treatment’ versus ‘pessary’ | 4 | Yes |
| 1. The decision aid should give the option ‘pessary’ versus ‘surgery’ | 5 | Yes |
| 1. The decision aid should give the option ‘surgery’ even if the patient never used a pessary | 5 | Yes |
| 1. Patients should have tried a pessary before they undergo surgery | 3 | No |
| 1. The decision aid should help patients to clarify their preferences | 5 | Yes |
| 1. The decision aid should end in a summary based on the given answers of the patients | 4 | Yes |
| 1. The decision aid should give a treatment advice based on the given answers of the patients | 3 | No |
| 1. The decision aid should not give a treatment advice based on the given answers of the patients | 3 | No |
| 1. The decision aid helps patients to define a treatment preference but does not give a treatment advice | 4 | Yes |
| 1. Patients are allowed to skip information/steps in the decision aid | 4 | Yes |
| 1. After reading the information, the knowledge and understanding of the information, should be tested with some test questions | 4 | Yes |
| 1. For patients with symptoms is ‘wait and see’ a good treatment option | 3 | No |
| 1. For patients with symptoms is a pessary a good treatment option | 5 | Yes |
| 1. For patients with symptoms is a surgery a good treatment option | 5 | Yes |
| **Risks and side effects (N=17)** |  |  |
| In the treatment option ‘wait and see’: |  |  |
| 1. The success rates of PFF should be mentioned | 4 | Yes |
| 1. The percentage of woman who will switch to a pessary or surgery should be mentioned | 4 | Yes |
| 1. The side effects that occur in less than 1% should be mentioned | 3 | No |
| 1. The risk of increased vaginal secretion should be mentioned | 5 | Yes |
| 1. The risk of sometimes some blood loss should be mentioned | 5 | Yes |
| 1. The risk of pressure spots in the vaginal wall should be mentioned | 5 | Yes |
| 1. The risk that the pessary may fall out should be mentioned | 5 | Yes |
| 1. The replacement and cleaning of the pessary every 3-6 months should be mentioned | 5 | Yes |
| 1. The daily 'confrontation' with the pessary should be mentioned | 3 | No |
| 1. The percentage of woman who will switch to surgery should be mentioned | 4 | Yes |
| 1. The percentage of woman who still wears the pessary after one year should be mentioned | 4 | Yes |
| In the treatment option ‘a surgery’: |  |  |
| 1. The risk of complications during and after the operation should be mentioned | 5 | Yes |
| 1. The advantages and disadvantages between uterine preservation and a hysterectomy should be mentioned (by a top of the vagina / uterine prolapse) | 5 | Yes |
| 1. The fact that a catheter is given for 1-2days after surgery should be mentioned | 4 | Yes |
| 1. The recovery period of six weeks should be mentioned | 5 | Yes |
| 1. The risk of a period of pain after the operation should be mentioned | 4 | Yes |
| 1. The percentage of woman which received a recurring prolapse after surgery should be mentioned | 5 | Yes |
| **Values clarification exercises (VCEs) (N=8)** |  |  |
| ‘Wait and see’ versus ‘a pessary’: |  |  |
| 1. ‘If a pessary is not really necessary, I would rather wait’ versus ‘my preference is a pessary even though it might not be necessary’ | 2 | Yes |
| 1. ‘I find the possible side effects of a pessary, such as vaginal secretion, hard to accept’ versus ‘I find the possible side effects of a pessary, such as vaginal secretion, acceptable’ | 4 | Yes |
| 1. ‘I find the replacement and cleaning of the pessary every 3-6 months hard to accept’ versus ‘the replacement and cleaning of the pessary every 3-6 months is no problem’ | 4 | Yes |
| ‘A pessary’ versus ‘a surgery’: |  |  |
| 1. ‘I find it hard to keep it calm for 6 weeks’ versus ‘I do not mind to keep it calm for 6 weeks’ | 4 | Yes |
| 1. ‘I find the replacement and cleaning of the pessary every 3-6 months hard to accept’ versus ‘the replacement and cleaning of the pessary every 3-6 months is no problem’ | 4 | Yes |
| 1. ‘I find the possible side effects of a pessary, such as vaginal secretion, hard to accept’ versus ‘I find the possible side effects of a pessary, such as vaginal secretion, acceptable’ | 4 | Yes |
| 1. ‘I'm anxious for a surgery’ versus ‘I am not anxious for a surgery’ | 4 | Yes |
| 1. ‘I find a 30% chance of recurrence of the prolapse within two years after surgery not acceptable’ versus ‘I find 30% chance of recurrence of the prolapse within 2 years after surgery acceptable’ | 4 | Yes |
| **Total** |  | 38/44 |
